# Unraveling novel mechanisms controlling heterosis in seeds: advances and biotechnological applications in crops

**DOI:** 10.1093/jxb/eraf400

**Published:** 2025-09-09

**Authors:** Sara Belcapo, Elise Réthoré, Eric Nguema-Ona, Ignacio Ezquer

**Affiliations:** Department of Biosciences, University of Milan, Milan, Italy; Plant Nutrition R&D Department, Centre Mondial d’Innovation of Roullier Group, Saint-Malo, France; Plant Nutrition R&D Department, Centre Mondial d’Innovation of Roullier Group, Saint-Malo, France; Department of Biosciences, University of Milan, Milan, Italy; University College Dublin

**Keywords:** Abiotic stress, cereal research, heterosis, hybrids, molecular technologies, seed quality

## Abstract

Heterosis refers to the superior performance of hybrids over their parents (inbred lines) in one or more characteristics. Hence, understanding this process is crucial for addressing food insecurity. This review explores the traditional genetic models proposed to explain heterosis and integrates them with emerging perspectives such as epigenetic studies and multi-omics approaches, which are increasingly used to investigate the molecular basis of heterosis in plants. We focus especially on the role of heterosis on seed quality traits in different plant species, considering seed development as one of the most important steps in the plant life cycle. We examine how heterosis enhances plant establishment, growth and seed yield in hybrids compared with their parental lines. We also review the involvement of this process in tolerance and resistance against environmental stresses such as drought, salinity, and heat. While this review primarily focuses on seed biology impact of heterosis, we also discuss recent studies highlighting the role of heterosis in other key factors, such as the rhizosphere microbiome. Finally, we consider the use of recent technologies to broaden the spectrum of crops suitable for hybrid breeding.

## Teaser

This review describes the latest discoveries about the molecular basis of heterosis and how this process is involved in seed quality traits.

## Introduction

### History of heterosis

The first observation of heterosis dates to the 18th century with the work of Kölreuter on tobacco plants (Kölreuter, 1761). This phenomenon was more extensively described by Charles Darwin in 1876, who noticed in a large variety of wild and cultivated species, that cross-pollinated plants displayed higher growth and fertility rates compared with their self-pollinated equivalents (Darwin, 1876). In 1908, Shull and East independently studied and applied heterosis to corn breeding (East, 1908; Shull, 1908), and Shull defined the word ‘heterosis’ for the first time in 1914 (Shull, 1948). The genetic bases of heterosis have been theorized through three different but complementary models: (i) the dominance hypothesis by Davenport in 1908, further developed by Bruce and Jones (Davenport, 1908; Bruce, 1910; Jones, 1917); (ii) the overdominance hypothesis by both East and Shull (East, 1908; Shull, 1908); and (iii) the epistasis hypothesis by Jinks and Jones in 1958 (Jinks and Jones, 1958). These models will be further detailed in the next section of this review.

After its discovery, the concept of heterosis was introduced in breeding processes, and this led to the first commercial hybrid maize variety being produced in the 1930s. From 1930 to 1980, the increase in yield in commercial maize (*Zea mays* L.) hybrids attributable to genetic improvement averaged 92 kg ha^−1^ year^−1^ (linear) in USA (Duvick, 1984). Almost 100% of maize seeds in USA are now hybrid. Hybrid exploitation strategy has been adopted in other key cereals like sorghum (in 1957), rice (in 1964), wheat (in 1982), barley (in 1960-70) or triticale (in 2012) (Longin *et al.*, 2012; Hochholdinger and Yu, 2025). However, in these self-pollinating crops, the production of hybrids is more expensive compared with maize, due to the need of inducing male sterility in the female parent. Since the 1960s, many technical and economic efforts have been deployed in wheat to induce male sterility (cytoplasmic male sterility, chemical hybridization agents, photoperiod–sensitive or thermo–sensitive male sterility); consequently, the production of hybrid wheat seeds is still very low worldwide (<1% of cultivated lands), as the associated costs of generation and innovation remain very high, and require substantial investment (Longin *et al.*, 2012; Gupta *et al.*, 2019). Better results have been obtained with rice, when in 1976, after 12 years of extensive research, the first commercial hybrid based on a stable cytoplasmic male sterility system was released in China. In 2012, the cultivated area of hybrid rice reached more than 20 million ha worldwide: China leading with 17 million ha, followed by India, Vietnam, and Bangladesh with 1.4, 0.7, and 0.7 million ha, respectively (Longin *et al.*, 2012).

Other major field crops have also benefited from hybridization, like sugar beet (Owen, 1945), sunflower (Leclercq, 1969), cotton (Basbag and Gencer, 2007), soybean (Fang *et al.*, 2023), and rapeseed (Frauen and Paulmann, 1999), and their hybrids now represent a major part of the seed market (Paril *et al.*, 2024).

In vegetables, the expression of heterosis depends on the species: some self-pollinated crops do not lose vigor by inbreeding (Wehner, 1999). For example, beans, pea, or lettuce have a relatively low sensitivity to inbreeding depression, which is a decline in fitness and vigor in the offspring of genetically related individuals. However, in other plant families like *Solanaceae* or *Brassicaceae*, the production of hybrids has been developed for eggplant, tomato, cabbage, and broccoli breeding over the 20th century (Sambandam, 1962; Yordanov, 1983; Wehner, 1999). Potato hybridization is still a challenge due to the difficulty of producing homozygous genotypes. This can be due to self-incompatibility effects or inbreeding depression, and it is still a major subject of research, since advances in the field may improve potato yield and its resistance to pathogens (Lindhout *et al.*, 2018).

## Heterosis in plants: main developmental, physiological and metabolic features improved

Heterosis is a biologically significant phenomenon that has been studied extensively in many plants and crop systems. Despite this, its use in agriculture was mainly restricted to cereals (particularly maize, rice, and sorghum). Historically, the aim has been oriented towards getting higher yields and better uniformity using F_1_ seeds. However, the improved traits of F_1_ plants can be visible at various stages of plant development. The major impact in developmental traits in hybrid research include seed biology and germination (detailed in the next section), root development, plant height, or reproductive organ size. For root development and rhizosphere, a recent review compiled the effects of heterosis on cereal roots (increased number of seminal roots, lateral root density, primary root length, cortical cell size, root/shoot ratio, etc.; Baldauf and Hochholdinger, 2023). In terms of shoot and reproductive development, maize hybrids show better-parent heterosis (i.e. the performance of the hybrid is measured with respect to the parent having a superior performance for a specific trait) in a wide number of growth parameters, namely tassel length, plant height, leaf width and length, kernel height, ear length, or cob weight (Flint-Garcia *et al.*, 2009). Some studies also reported contrasting responses between vegetative and reproductive growth. In studies using tomatoes, plant height was found to be negatively affected by heterosis, while fruit yield per plant and fruit weight were improved (Tamta and Singh, 2018). In tomato, early fruit maturity is also a key factor that is considered in breeding programs (Liu *et al.*, 2021).

In addition to growth and yield parameters, heterosis can enhance specific traits related to product quality. For example, in rapeseed, the accumulation of fatty acids in grains is a major trait that determines its value and suitability for the edible oil industry. Different studies have examined the inheritance of fatty acid profiles and glucosinolate content in rapeseed (Ze-Su *et al.*, 2012; Tahir *et al.*, 2018; Niemann *et al.*, 2020). In sunflower, heterosis for oil yield and quality has also been assessed (Aslam *et al.*, 2010; Ahmed *et al.*, 2021). In other crops, quality traits can relate to specific parameters, such as protein content in bread wheat (Singh *et al.*, 2014), rice milling recovery and cooking properties (Shi *et al.*, 1997; Rukmini *et al.*, 2014), or cotton fiber quality (Bolek *et al.*, 2010).

As well as assessing morphological and qualitative traits, different studies have tried to understand heterosis at the physiological level. As a fundamental role in plants, photosynthesis has been shown to be a target of improvement in diverse crops (maize, cotton, sorghum) (Mehta and Sarkar, 1992; Chen *et al.*, 2005; Zhao *et al.*, 2024). Another major function that conditions plant growth is the nutrient uptake and use efficiency (e.g. NUE: Nitrogen Use Efficiency, PUE: Phosphorus Use Efficiency, KUE: Potassium Use Efficiency). In the last decade, several studies highlighted the beneficial effect of heterosis on nitrogen, phosphorus, or potassium uptake (Wei *et al.*, 2018; Wang *et al.*, 2019; Santos *et al.*, 2023). For example, an increase of mineral uptake between 12–20% was reported in rice hybrid lines compared with their inbred counterparts (Wei *et al.*, 2018).

To explain the regulation of shoot growth by heterosis, the role of hormones, particularly gibberellins, has been analyzed in hybrid lines compared with their parental lines across various cereal crops (maize, rice, wheat and sorghum) (Rood *et al.*, 1988, 1992; Zhang *et al.*, 2007; Ma *et al.*, 2011). Higher endogenous levels of gibberellins were detected in most hybrids compared with their relative inbreds, while the parental lines displayed an enhanced response to exogenous application of gibberellins (Ma *et al*., 2011). Another major hormone involved in the control of plant growth, auxin, was also shown to rapidly accumulate in hybrid seedlings; interestingly, this was correlated with a concomitant increase in palisade cell number and size in rapeseed (Zhu *et al.*, 2020). On the contrary, salicylic acid levels were down-regulated in Arabidopsis hybrids, and led to decreased expression of senescence-related genes, thereby extending photosynthetic activity in the leaves (Zhang *et al.*, 2016; Gonzalez-Bayon *et al.*, 2019; Smith, 2019). Repression of the ethylene biosynthetic pathway was also seen in Arabidopsis hybrids in comparison to their parents, and exogenous application of ACC (1-aminocyclopropane-1-carboxylic acid), a precursor of ethylene, decreased the heterosis effect (Song *et al.*, 2018; Birdseye *et al.*, 2021).

At the proteome level, in inbred lines of different species, a higher proportion of unstable proteins was produced, demonstrating that energy and resources are diverted from biomass accumulation towards maintaining protein homeostasis (Goff, 2011). Other studies suggest that specific proteins are accumulated in hybrid lines compared with their parents. In Arabidopsis, it was recently reported that plant height heterosis is linked to a higher accumulation of plastid ribosomal proteins (Birdseye *et al.*, 2021). Surprisingly, a lower correlation was found for the genes associated with photosynthesis. In a recent analysis, the accumulation of growth-promoting and energy metabolism-related proteins in elite hybrid rice was highlighted (Ma *et al.*, 2023a). Mid-parent heterosis (i.e. when the performance of the hybrid is measured with respect to the average of its two parents) negatively correlated with stress-responsive proteins, but positively correlated with proteins involved in translation, catabolism, carbohydrate metabolism and reproductive development. Increased lysine acetylation and histone acetylation of proteins present in the hybrids were also identified as modulators of energy metabolism and growth (Ma *et al*., 2023a). Another comparative proteomic analysis reported an important increase in the accumulation of proteins related to photosynthetic activity (light reactions, Calvin cycle and photorespiration) and stress-responsive proteins (secondary metabolism, proteolysis, TCA, redox state, or abiotic stress) in two maize hybrids compared with their parents (Wang *et al.*, 2021a). Overall, data show varying trends: some show similar effects, while others reveal contrasting outcomes on the impact of heterosis on stress-responsive protein accumulation, reflecting the complex genetic and epigenetic models behind hybrid effects on traits. The multifactorial nature of heterosis continues to pose a significant challenge for breeders and geneticists. Further molecular, developmental, cell biological, and agronomic research is needed to better integrate the physiological changes regulated by heterosis.

## Genetic bases of heterosis and epigenetic modifications involved

### Genetic models describing heterosis

The complexity of heterosis likely arises from the diverse genetic and epigenetic interactions contributing to hybrid performance, coupled with challenges in clearly defining and categorizing its various subtypes in plants.

**Fig. 1.**
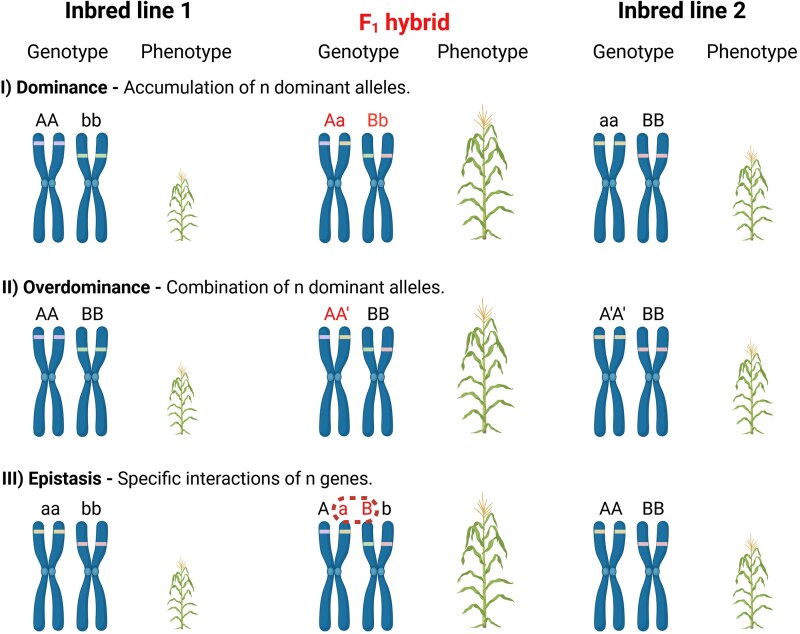
Heterosis genetic models for dominance, overdominance, and epistasis hypotheses. Schematic representation of the genetic bases of heterosis described by the three classical models: (I) the **dominance hypothesis** explains the better phenotype of the F_1_ hybrid (Aa/Bb) compared with the parental lines, with the complementation of the recessive deleterious alleles (present in the parents: AA/bb and aa/BB) by the dominant superior alleles; (II) the **overdominance hypothesis** identifies the cause of the F_1_ hybrid (AA’) superior trait in the combination of two dominant superior alleles from the maternal and paternal lines (AA and A’A’); (III) the **epistasis hypothesis** describe the better performance of the F_1_ hybrid at a genetic level with the interaction between A and B loci (in the case represented on the picture, a and B). Created in BioRender. Ezquer, I. (2025) https://BioRender.com/rip8tsd.

The use of molecular markers to predict heterosis represents a major step towards identifying key genomic regions associated with hybrid performance and supporting precision breeding strategies. Overall, three key hypotheses have been put forward on the genetic mechanisms behind heterosis: (i) dominance; (ii) overdominance; and (iii) epistasis (Fig. 1). It is important to clarify that the terms hereby mentioned as ‘dominance’ and ‘overdominance’ are used as models explaining the mechanistic effect of heterosis as referred to genome-wide mechanisms of hybrid vigor, therefore not as patterns of trait inheritance at individual loci for specific traits. These mechanisms are influenced by parental type, trait nature, genetic and epigenetic interactions, environmental factors, chromosomal compatibility, gene dosage effects, and inheritance of organelles such as mitochondria and plastids (Groszmann *et al.*, 2013; Hochholdinger and Baldauf, 2018; Wu *et al.*, 2021; Paril *et al.*, 2024). Each hypothesis (briefly described below) has its own implications for breeding and crop improvement, and they are often considered in combination, rather than in isolation, since heterosis likely results from effects of multiple genetic interactions.

The **dominance** hypothesis suggests that heterosis arises from the masking of deleterious effects associated with recessive alleles, by their corresponding dominant alleles. In this model, hybrids benefit from the suppression of negative traits that would otherwise impair growth or fitness, if the recessive alleles were present in a homozygous state in one or both parents. Mechanistically, recessive alleles in each parent may contribute to suboptimal phenotypes or reduced performance when homozygous. In hybrid alleles, the combination of genetically distinct parents results in heterozygosity, where dominant alleles effectively ‘mask’ the adverse effects of recessive alleles. The **overdominance** hypothesis postulates that heterozygosity at specific loci confers phenotypic advantages superior to those of homozygous parents. This advantage arises from the optimal interaction of combinations of heterozygous alleles, which improves the function or increases the resistance of crops. **Epistasis** occurs in cases of interactions between genes, where the effect of one gene is modified by one or more ‘modifier’ genes. These interactions can generate phenotypes that outperform the effects of individual genes. In epistatic hybrids, allele combinations from two parents can generate novel epistatic interactions, enhancing traits like growth or yield beyond both parents’ potential.

As an example, in a study evaluating 1253 hybrid maize populations for key agro-quality traits — including grain moisture, silking date, plant height, and grain yield — Quantitative Trait Loci (QTLs) analysis revealed distinct genetic patterns underlying these traits (Larièpe *et al.*, 2012). Many QTLs associated with grain yield exhibited strong overdominance effects, with minimal differences observed among heterozygous genotypes. In contrast, grain moisture was primarily influenced by additive genetic effects, suggesting limited heterotic contribution. For plant height and silking date, the observed genetic effects were intermediate, reflecting a mix of additive and dominance contributions. Recent research evaluated maize hybrids to validate QTLs for tunnel length (a key indicator of borer infestation and eventually, the effectiveness of the resistant strategies used) and grain yield, using a MAGIC (Multiparent Advanced Generation Inter-Cross) population (López-Malvar *et al.*, 2024). Maize hybrids were obtained by crossing recombinant inbred lines (RILs) with the A638 tester. The study highlighted that dominance significantly influenced grain yield, with lower QTL validation rates than tunnel length, due to dominance and epistasis masking additive effects. In fact, epistasis complicated the validation of yield QTLs, while in simple traits such as tunnel length, it was less relevant.

Dominance has recently been reported in rice, where hybrids have been characterized by significantly higher yields than the parental lines (Wang *et al.*, 2024a). Authors reported dominance effects in traits such as disease resistance, where recessive alleles causing susceptibility are masked in hybrids. A canonical example of overdominance is demonstrated by the *SINGLE FLOWER TRUSS* (*SFT*) gene in tomato (Krieger *et al.*, 2010). The study reported that heterozygosity for a functional *SFT* allele alongside a loss-of-function allele resulted in superior yield performance due to overdominance. Similarly, in *Arabidopsis thaliana*, the *FLOWERING LOCUS T* (*FT*) gene, a homolog of *SFT*, regulates florigen synthesis (Lifschitz *et al.*, 2006, 2014). When heterozygous, *FT* promotes the differentiation of more inflorescences compared with the parental lines, exhibiting an overdominance effect that enhances heterosis.

Recent studies on tomato hybrids between *Solanum lycopersicum* cultivars ‘Micro-Tom’ and ‘M82’ reported overdominance effects, exhibiting a yield like that of ‘M82’, although the vegetative biomass was reduced (Rajendran *et al.*, 2022). Notably, an overdominance effect was observed in the number of harvested fruits, suggesting that hybrid vigor can be utilized for yield improvement. The authors also identified that specific ‘Micro-Tom’ mutants with floral and size defects could be used to screen for mutants that induce overdominance effects in F_1_ hybrids, enhancing the potential for field productivity.

Multiple epistatic events have been reported so far in breeding programs in both cereals and dicots (see Dwivedi *et al.*, 2024). In maize, digenic epistatic interactions were reported to contribute to grain yield and its components. For instance, in maize, QTL KNPR6 has been identified as a key player in the control of yield by affecting kernel number per row (KNPR) (Liu *et al.*, 2012). More recently, seven SNPs (Single-Nucleotide Polymorphisms) in maize were discovered to be located in QTL yield traits. Interestingly, among the candidate genes, some of them were already identified as regulators of seed development. Moreover, it was also reported that the gene *Zm00001d016656*, which encodes for a serine/threonine protein kinase, associated with five different traits across multiple environments (Zhang *et al.*, 2022).

In addition, digenic epistatic interactions include additive×additive effects and interactions with the environment. Epistasis plays a central role in modulating effects of QTLs, suggesting that many loci influence traits primarily through interactions, rather than direct effects (Dwivedi *et al.*, 2024). In barley, epistasis was reported as a major effector for the genetic control of traits like plant height and seed weight (Ma *et al.*, 2007). In rice, multiple studies on epistasis have been reported, and excellent reviews summarize them (Dwivedi *et al.*, 2024). For example, in bell pepper (*Capsicum annuum*), epistasis influenced resistance to stem blight (Bartual *et al.*, 1993). In groundnut (*Arachis hypogaea*), epistasis characterized traits related to pod yield in different environments, and nutritional quality by protein and oil content (Upadhyaya and Nigam, 1998). In common bean (*Phaseolus vulgaris*), epistasis—especially when combined with linkage disequilibrium (a situation where certain alleles are inherited together more often than expected by chance)—can introduce bias in the prediction of yield and seed weight, with more significant effects in crosses between gene clusters (Borel *et al.*, 2016). These cases illustrate the complexity of epistasis in crop breeding, and its impact on trait prediction and genetic improvement.

### Epigenetic modifications in heterosis

The genetic basis of heterosis is complex and often does not correlate with the genetic distance between parents. Remarkably, high levels of heterosis can occur even among closely related parents with minimal genetic distance. This phenomenon has been demonstrated in studies exploiting natural *Arabidopsis thaliana* ecotypes (Groszmann *et al.*, 2014; Greaves *et al.*, 2015; Kawakatsu *et al.*, 2016; Lang *et al.*, 2016). Arabidopsis remains a fantastic model for this, since researchers have advanced omics tools, such as those provided by the 1001 Genomes Project (https://1001genomes.org/). However, despite a high degree of DNA sequence similarity (approximately 96%), hybrids between Arabidopsis ecotypes show significant increases in biomass and seed yield (Greaves *et al.*, 2015). This enhanced vigor is attributed to the interaction of closely related genomes with differing parental epigenomes, leading to novel transcriptional patterns in the hybrids.

Another important current research field related to epigenomics is chromatin accessibility. Recent studies have described tea and rice hybrids possessing a larger number and length of accessible chromatin regions (ACRs) with respect to the parents, likely resulting in a stronger transcriptional activity of target genes (Wang *et al.*, 2022, Wang e*t al*., 2024b). In particular, in a study on tea plants (*Camellia sinensis*), the assay for transposase-accessible chromatin with sequencing (ATAC-seq) was used; that is a simple and rapid method based on the sensitivity of non-specific nuclease cleavage applied to identify accessible chromatin regions (Wang *et al.*, 2022).

These findings underscore the pivotal role of epigenetic changes in driving hybrid vigor. Several epigenetic modifications have been reported to contribute to heterosis in plants, as highlighted in multiple reviews (Greaves *et al.*, 2015; Kumar and Rani, 2023; Kakoulidou *et al.*, 2024; Kakoulidou and Johannes, 2024).

#### DNA methylation

Altered methylation patterns, including CG, CHG, and CHH methylation, have been seen in hybrids. These changes influence gene expression and phenotypic outcomes, playing a critical role in heterosis. Multiple examples have been described in the literature (see Table 1; Kakoulidou and Johannes, 2024). Recently, methylation and gene expression patterns were analyzed in two geographically distinct populations of wild emmer wheat (*Triticum turgidum ssp. dicoccoides*) and their F_4_ hybrids (Ziv and Kashkush, 2024). The study reported that wheat F_4_ hybrids exhibited reduced methylation and increased gene expression compared with their parental lines, with differentially expressed genes (DEGs) linked to stress response, photosynthesis, and environmental adaptation. Key wheat hybrid vigor traits, such as intermediate seed yield, greater plant height, and enhanced photosynthetic gene expression, were observed, although chlorophyll content remained unchanged. The authors proposed the use of the genetic diversity of wild emmer wheat as a powerful tool for future breeding programs that use hybridization and SNP-based marker-assisted selection to improve wheat resilience and productivity.

#### Histone modifications

Specific histone modifications are associated with transcriptional regulation in hybrids: activating modifications include acetylation of histones H3 and H4 (H3ac and H4ac), and trimethylation at lysine 4 and lysine 36 of H3 (H3K4me3 and H3K36me3). Repressive modifications, such as H3K9me2 and H3K27me3, regulate gene expression related to heterosis. Hybrid-specific histone modifications have been found, emphasizing their importance in heterosis mechanisms (Ma *et al.*, 2023b; Kakoulidou and Johannes, 2024). In a recent study in *Brassica napus*, the molecular basis of heterosis in allopolyploidy was investigated by analyzing epigenome and transcriptome data of an elite hybrid and its parental lines in multiple tissues (Ma *et al.*, 2023b). Transcriptomic and epigenetic approaches showed that variations in H3K4me3 (associated with transcriptional activation) and H3K27me3 (linked to repression) were observed between the parents and the hybrid, mainly reflecting parental differences. Both H3K4me3 and H3K27me3 modifications were relatively stable in the hybrid, and were inherited additively. Overall, this study underlined the value of transcriptome changes and remodeling of histone modifications in understanding heterosis in allopolyploid crops, offering valuable insights for crop breeding strategies.

#### Small RNA (sRNA) regulation

Small RNAs are vital for post-transcriptional regulation. They inhibit translation or target specific mRNA transcripts (see Table 1; Kakoulidou and Johannes, 2024). A recent study explored the role of small RNAs (sRNAs) in hybrid vigor in cereals (Crisp *et al.*, 2020). The study analyzed variation and inheritance in a broad maize population, including five tissues, eight inbred parental lines, and 12 hybrid genotypes (Crisp *et al.*, 2020). The authors reported that sRNA expression varies significantly across developmental stages, genotypes, and types (21-nt, 22-nt, and 24-nt). The maize genome exhibits a developmentally plastic and complex distribution of sRNAs, particularly for 22-nt sRNAs. In hybrids, sRNAs demonstrate locus-specific inheritance patterns, predominantly partial or complete dominance, which rarely exceeded parental ranges, meaning that hybridization has effects on sRNA expression at specific loci, not on a global scale. The authors elegantly highlight the important role of sRNAs in cereal heterosis, and provide a valuable resource for advancing plant breeding strategies. Another study assessed the impact of allopolyploidization on sRNA profiles in a diploid potato hybrid and its allopolyploid offsprings (Barber *et al.*, 2012). The authors reported that the genomic impact from whole genome duplication led to a regulated pattern of rearrangement of sRNA profiles (understood here as structural reorganization of RNA at primary or secondary structure). Analysis of differentially accumulated RNAs showed that 24 nt RNAs mapped to exons correlated with gene expression changes, and these findings emphasize the non-random nature of RNA rearrangement after polyploidization (Barber *et al.*, 2012). The study suggests that transposons and sRNAs play a significant role in regulating genetic variation, and contribute to the heterosis observed in maize.

MicroRNAs (miRNAs) and long non-coding RNAs (lncRNAs) regulate gene expression and contribute to heterosis traits by modulating transcriptional and post-transcriptional processes. For a complete and updated overview on the emerging role of ncRNAs in regulating plant growth, development, and stress responses see Yadav *et al.* (2024). NcRNAs, including small interfering RNAs (siRNAs), miRNAs, lncRNAs, and circular RNAs (circRNAs), function beyond traditional RNA roles, influencing transcription, translation, and epigenetic processes. NcRNAs play a key role in plant resilience to environmental stresses such as drought, salt, flooding, heat, and cold (Gill *et al.*, 2024), demonstrating that this field of research is critical to address plant adaptation.

Orantes-Bonilla *et al.* (2023) recently investigated the molecular mechanisms controlling heterosis in *Brassica napus* (oilseed rape) hybrids. They combined transcriptomic and epigenomic changes during seed and seedling development, revealing new insights into gene expression and methylation patterns. While differential expression was observed in ∼30 000 genes, >300 microRNAs, and 35 000 sRNAs, the authors also identified >7000 differentially methylated regions. Of these, ∼70% exhibited parental dominance, with the patterns of the hybrid aligning with one of the parent lines. Interestingly, maternal dominance was more prominent during seed development, contrasting with the demethylation typically seen in maternal gametes during gametogenesis. Overall, this study on *Brassica napus* exemplifies how hybrids exhibit robust plant architecture and increased seed weight compared with parents, supporting the functional significance of identified molecular patterns and relationships at multiple levels (expression and epigenetic), and offering new perspectives for improving hybrid breeding strategies.

## Heterosis at seed stage in plants

### Main seed traits improved with heterosis

**Fig. 2.**
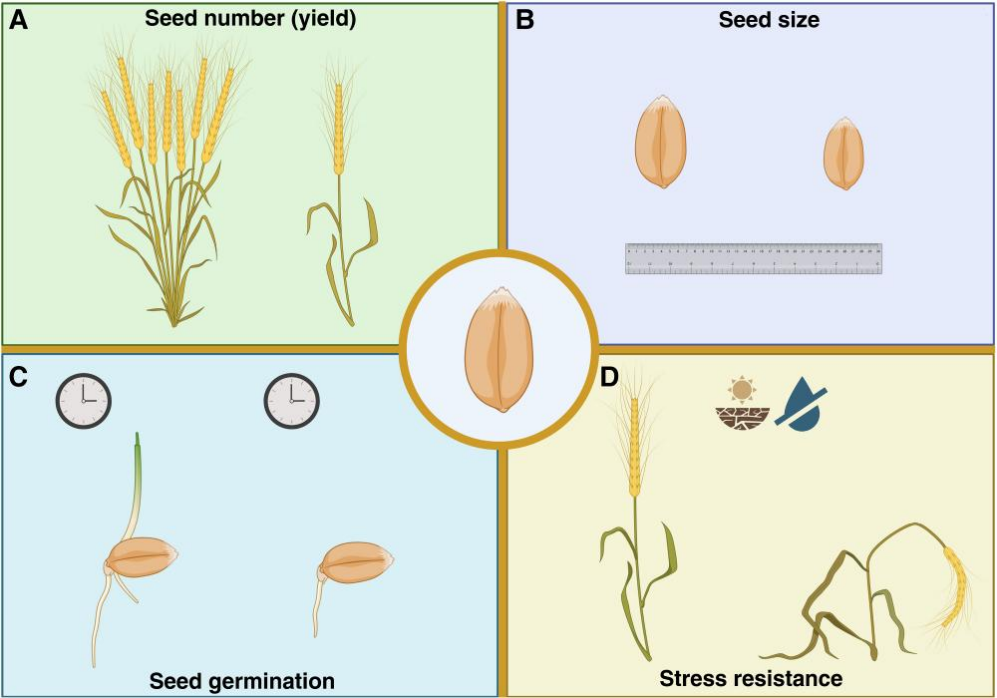
Seed traits affected by heterosis. Heterosis is a complex process involved in different traits at different developmental stages of the plant. At seed stage, it has been shown that heterosis can impact: (A) **seed yield** by increasing it; (B) **seed size** by leading to the production of bigger seeds; (C) **seed germination** by obtaining seeds with a reduced germination time; and (D) **response to environmental stresses** by making seeds more tolerant/resistant. Created in BioRender. Ezquer, I. (2025) https://BioRender.com/8zwpip9.

Heterosis has been observed not only for traits in adult plants, but also in early stages like those affecting seedling (Hoecker *et al.*, 2006) and seed development (Meyer *et al.*, 2007; Wan *et al.*, 2022). For seed development, there are different traits related to heterosis at this stage; from the number of seeds per plant (related to grain yield) to germination and stress resistance. Listed below are those examined, and the most studied in the literature (Fig. 2). This review focuses on heterosis at the seed stage, as seeds play a crucial role in plant life cycles, ensuring generational survival.

Moreover, heterosis has been largely studied and described in crop species, especially maize and rice, but also in other model plant systems such as *Arabidopsis thaliana* (Castillo-Bravo *et al.*, 2022), providing useful tools for genetic improvement of these traits in many other crops systems. Hence, seed traits and heterosis have been studied in distinct species, and we here include a general overview of recent advances.

#### Seed yield

Seed yield (or grain yield) is a quantitative trait dependent on different plant components (number of fertile tillers, spikelets per panicle, florets per spikelet) and seed characteristics (number of seeds, seed weight, and size). The achievable seed yield is also determined by several other factors such as genetic traits, breeding activities, and climate conditions. Heterosis in major food crops, including maize and rice, has greatly improved grain yield in the past (Virmani *et al.*, 1982; Duvick, 2001).

Heterosis is considered the main contributing factor for the success of plant breeding projects related to major crop species to achieve early development, high-yielding and uniform cultivars which combine the most important economic characteristics (Avdikos *et al.*, 2021). In maize, heterosis is one of the main factors involved in hybrid performance, especially for grain yield (Li *et al.*, 2021). In fact, maize is the most successful crop in which heterosis highly improved agricultural production over decades (Hochholdinger and Baldauf, 2018). Another important cereal where heterosis has been studied and characterized is rice. A recent report showed an increase in grain yield of restorer lines (increased by approximately 34.1–47.5% compared with the corresponding inbred rice cultivars) by inducing an alternative splicing event in a heterosis gene, *OsMADS1* (MINICHROMOSOME MAINTENANCE 1, AGAMOUS, DEFICIENS and SERUM RESPONSE FACTOR), via CRISPR-Cas9; this resulted in a different protein associated with the increase in grain length and weight (Wang *et al.*, 2024a). Furthermore, in tomato, different studies have reported an increase in fruit number and yield of nearly 60%, and even higher in specific cases, due to the use of hybrids (Hannan *et al.*, 2007; Ahmad *et al.*, 2011; Avdikos *et al.*, 2021).

#### Seed size

Seed size is a contributing factor in determining grain weight, and therefore ultimately influencing yield (Cui *et al.*, 2011; Patil *et al.*, 2013; Mohammadi *et al.*, 2021; Qu *et al.*, 2021). In bread wheat, kernel weight contributes to ∼20% of the genetic variation in grain yield (Schierenbeck *et al.*, 2021), and also plays a role in the determination of other quality factors (i.e. test weight, flour yield and milling quality) (Tyagi *et al.*, 2015; Wang *et al.*, 2021b). Moreover, different studies suggested a positive influence of larger kernels on seedling vigor and early growth in different crops, as in rice and bread wheat (Avni *et al.*, 2018; Sun *et al.*, 2020). Therefore, studying this trait, which consists of various parameters such as area, perimeter, length, and width, is necessary.

The relationship between heterosis and seed size has also been analyzed in non-crop species, such as *Arabidopsis thaliana*, which, due to its large number of genotyped accessions and high natural genetic diversity (Weigel, 2012), is considered an excellent tool for examining parental genotype combinations that induce heterosis effects (Stokes *et al.*, 2007; Schnable and Springer, 2013). Key genes identified for instance in GWAS (genome-wide association studies), like those for improving seed size and yield, can be used in breeding programs for other crops. Moreover, several Arabidopsis intraspecific hybrids generated over the years have already shown heterosis impact on seed size (Alonso-Blanco *et al.*, 1999; Stokes *et al.*, 2007; House *et al.*, 2010; Groszmann *et al.*, 2014; van Hulten *et al.*, 2018; Wang *et al.*, 2020), making this model plant system optimal to better understand the control of this process. The influence of the maternal and paternal genomes on F_1_ hybrid seed size, as well as the parental effect on F_1_ seed size heterosis has been studied (Castillo-Bravo *et al.*, 2022). The results showed that interactions between genetic hybridity and parental genome dosage can amplify heterosis effects in plants. The study also reported that maternal effects on F_1_ seed size can be both sporophytic and gametophytic, while the paternal effects are likely gametophytic, suggesting overall that maternal and paternal contributions to F_1_ seed size are independent, and governed by different genetic pathways. Interestingly, the authors reported that genetic hybridity in inter-ploidy crosses can significantly amplify parent-of-origin effects on seed size heterosis, proving that parental genome dosage influences hybrid vigor in seed size. This is a concrete example of how the integration of genomic tools, including the 1001 Genomes Project in *Arabidopsis thaliana* (https://1001genomes.org/), biodiversity studies, and access to genotyped populations, can bridge basic and applied sciences. These resources facilitate the identification of previously unknown gene candidates, not only enhancing our fundamental understanding of genetic variation, but also providing molecular clues for breeding programs focused on improving heterosis in other non-model crops.

#### Seed germination

Seed germination is a fundamental and indispensable step in the plant life cycle that marks future growth and development. It plays a key role in the development of subsequent seedling emergence, in plant establishment and vigor, in stress tolerance, and in flowering time, and therefore further affects total yield and productivity (Carrera-Castaño *et al.*, 2020; Al-Khafaji *et al.*, 2024). In relation to this, climate change alterations pose significant challenges to both agriculture and biodiversity by disrupting seed germination patterns. In crops, extreme temperatures, irregular rainfall, and soil degradation can reduce germination rates, leading to lower yields and threatening food security (HLPE, 2023).

Germination is tightly coordinated among the different seed organs (seed coat, embryo, and endosperm) and is characterized by three different phases. During the early stage of germination the inactive dry seed rapidly imbibes water. This is followed by metabolic reprogramming within the seed, and subsequent energy mobilization from reserve tissues (like endosperm) to zygotic tissues activating embryo growth. The process involves a finely balanced interplay among several transcription factors, enzymes, hormones, and cell wall homeostasis, ultimately leading to the protrusion of the radicle from the seed coat and triggering seedling establishment (Nonogaki *et al.*, 2010; Ezquer *et al.*, 2016; Paolo *et al.*, 2021; Di Marzo *et al.*, 2022).

A critical factor in successful germination is the management of reactive oxygen species (ROS), which are generated during seed rehydration, and represent a major source of cellular damage. Germination depends on maintaining an optimal oxidative window and the ability of cells to repair DNA damage accumulated during seed development, maturational drying, and germination (Sincinelli *et al.*, 2025).

Seed germination is a complex process, particularly because it is influenced by both intrinsic (such as seed dormancy and available food stores) and extrinsic factors (such as water, temperature and light) (Miransari and Smith, 2014). Moreover, it is recognized as a fundamental trait associated with high heterosis, necessary for the establishment of healthy seedlings, and therefore related to plant vigor (Wan *et al.*, 2022). Previous studies have proved that F_1_ hybrid seeds displayed enhanced seed germination performance relative to their inbred parental lines (Li *et al.*, 2016). It has also been reported that embryo expansion during the initial stages of maize seed germination is a characteristic pattern of heterosis; analysis of this growth process at a proteomic level revealed that the major difference in hybrids compared with the parents was an increase in the amount of non-additive proteins, including HSP20, a heat shock protein, that correlate with heterosis appearance during germination (Ouyang *et al.*, 2009; Fu *et al.*, 2011).

Water uptake in seed germination occurs in three different phases: seed imbibition and reactivation of metabolic process, followed by a lag phase of water uptake involving cellular and metabolic adjustments, and finally, the mechanical emergence of the radicle accompanied by further water uptake to support cell expansion (Finch-Savage and Leubner-Metzger, 2006). During phase two, the nutritional support to the growing embryo derives from the seed's own reserves until phase three. This implies that in the last phase, both genetic and environmental cues determine the germination potential of the seed (Holdsworth *et al.*, 2008), while in the second phase, the embryo tissue is unaffected by external influences, and hence provides a useful system to explore heterosis (Fu *et al.*, 2011).

Recently, several studies have focused on investigating the contribution of the endophytic microbiome to heterosis. In fact, although the majority of the articles currently available in the literature are focused on elucidating the genetic mechanisms of the basis of heterosis, there is recently an increasing body of evidence supporting the idea that the plant microbiome is a major element towards understanding hybrid vigor (Wagner *et al.*, 2021; Liu *et al.*, 2024a). The first recent study exploring this hypothesis demonstrated that the hybrid vigor/root biomass ratio in maize significantly depends on the rhizosphere microbiome (Wagner *et al.*, 2021). The rhizosphere is influenced by root exudates, which recruit and host diverse beneficial microbes that promote plant growth and the ability to tolerate biotic and abiotic stresses. Importantly, many of these microbes can be transmitted to seeds, thus conferring beneficial traits to progeny (Araujo *et al.*, 2024). A recent study investigated the role of the endophytic microbiome in heterosis at the seed stage in rice (Liu *et al.*, 2024a). In particular, the authors analyzed how hybridization affects the bacterial and fungal communities of seeds, and verified whether this promotes higher seed germination. They found differences in the diversity and abundance of microbial taxa mainly associated with plant growth, both being higher in seeds of rice hybrids than the parental lines. In these studies, they concluded that hybridization directly influences the composition of both rhizosphere and seed endophytic microbiomes (Wagner *et al.*, 2021; Araujo *et al.*, 2024; Michl *et al.*, 2024), and therefore, that a portion of the changed microbiome resulting from hybridization can be vertically transmitted from the rhizosphere to seed, positively affecting the progeny.

#### Environmental stress tolerance/resistance

Environmental stress is defined as the interaction between external stressors and a biological system, leading to a detrimental change in the structure or function of the system, potentially threatening its survival (Cairns, 2013).

##### Drought stress

One of the environmental stresses most studied is drought, a water-deficit condition that mainly leads to plant cellular dehydration and a concomitant increase in osmotic pressure. This triggers physiological and biochemical alterations such as plasma membrane damage, inhibition of photosynthesis, and ROS accumulation (Dai *et al.*, 2024). One of the criteria for determining drought stress resistance in a plant is the capacity to recover from the damage after re-watering (Dai *et al.*, 2024), but in some cases strong and prolonged drought can even lead to cell death.

Plants show different drought stress phenotypes that vary according to the developmental stage (e.g. stunted seedling growth, rolling leaves, pollen abortion, suppression of seed-filling, and reduction in grain yield) (Zenda *et al.*, 2018; Hu *et al.*, 2020; Bheemanahalli *et al.*, 2022; Liu *et al.*, 2023; Dai *et al.*, 2024). Seed germination is one of the most sensitive growth stages to water deficit (Surbhaiyya and Wagh, 2018).

Seeds are essential because through germination, they produce and ensure success of the next generation. Hence, it is not surprising to discover that plants have acquired mechanisms to protect seeds. Once they are harvested from the plant, seeds are in a dry, quiescent state where they are protected by the seed coat that allows them to be more tolerant to stress factors (i.e. high temperatures) compared with other plant development stages, such as adult plants. In contrast, during seed development or during germination, seeds are very susceptible to stress (Kranner *et al.*, 2010). In particular, drought is most responsible for global crop yield loss, and also the related impact on germination can lead to vigor loss or reduced viability. In fact, during this process, metabolic reprogramming involving hydrolytic and synthesis of new, specialized metabolites require hydration, and the absence or reduction of water at that critical step may affect the speed and uniformity of germination, likely resulting in low rates in plant establishment and reproduction (Kranner *et al.*, 2010).

Many hybrids have been analyzed for displaying greater resistance, compared with their parents, to environmental stresses such as drought, due to heterosis. Also in this context, a significant body of research has been conducted on maize. Seed germination and different seedling phenotypes of maize genotypes have been studied under drought stress, in order to select the most promising ones to then use as parents to produce tolerant hybrids, which can be exploited in breeding programs (de Abreu *et al.*, 2019). The authors observed that different traits (seedling emergence, emergence speed index, shoot length, root length, number of seminal roots, shoot dry weight, root dry weight and ratio between root and shoot length) displayed a better performance in the studied hybrids compared with their parents in both stress and non-stress conditions, showing a significant heterosis expression (de Abreu *et al.*, 2019).

As mentioned earlier, drought stress also affects seed maturation on the mother plant, and thus yield. In fact, insufficient water availability can cause important alterations of the physiological processes leading to major negative effects in plant growth and yield stability: in 2019 it was estimated that yield losses were between 30–90% due to drought stress, depending on the crops (Dietz *et al.*, 2021). In 2024, Dai and colleagues reported the different impact of drought conditions on parental lines and maize hybrids. To uncover the impact of drought on heterosis, the authors cultivated hybrids and specific parental inbred lines to test the performance under well-watered and water-deficit conditions, and then investigated different traits related to plant growth and production (such as length and grain yield). Overall, the authors reported that hybrids show reliable heterosis under both normal and drought conditions (Dai *et al.*, 2024).

Regarding drought tolerance and resistance, other crops have been studied. In kenaf (*Hibiscus cannabinus* L.), for example, seedling parent lines and hybrids treated with PEG6000 (to mimic drought stress) displayed relevant heterosis in F_1_ under drought conditions (Luo *et al.*, 2023).

##### Heat stress

Another environmental stress that highly affects plant growth is heat stress. High temperatures can cause damage at every stage of plant development, but some of them are more sensitive to heat than others (Jagadish *et al.*, 2021). Among the different developmental periods, early establishment, flowering, and gametogenesis are the ones most affected by heat (Wahid *et al.*, 2007; Jagadish, 2020), particularly in crops, where heat stress can result in high yield loss. Among other effects, an increase in temperature leads to the production of ROS in plant tissues (Suzuki and Katano, 2018; Huang et al., 2019). These molecules can play an important signaling role in the response to stress (Mittler, 2017), including heat stress, and controlling their levels is crucial for stress tolerance.

Analysis and characterization of hybrids that can better adapt and survive at higher temperatures has been performed. In spring canola (*Brassica napus* L.), it has been shown that heat treatment caused a 20% and 25% reduction in seed yield within the hybrids and inbreds, respectively (Koscielny *et al.*, 2018). This indicates that heterosis can reduce the impact of higher temperatures on canola yield.

##### Soil salinity stress

Among the environmental stresses that affect plant growth, soil salinity is one of the most problematic for sustainable agriculture (Zhao *et al.*, 2020). It has negative effects on different stages of plant development, such as seed germination, crop growth, and productivity (Yang and Guo, 2018; Liu *et al.*, 2022). High salinity can damage plants in several ways: accumulation of Na^+^ and Cl^−^ in cells that reduce the uptake and transport of K^+^ and Ca^2+^, which affects enzyme activities, ROS accumulation, and osmotic stress that impairs nutrient and water uptake by roots (van Zelm *et al.*, 2020; Zhao *et al.*, 2020; Dai *et al.*, 2022; Liu *et al.*, 2022). Furthermore, several studies have reported the effects of salinity stress on plant development. In *Brassica napus* L., the levels of nitric oxide (NO), a signaling molecule involved in several physiological functions, were analyzed during plant growth and stress response, in both parental lines and related hybrids. A higher germination rate was reported, corresponding with a significantly higher quantity of NO, in the hybrids than in the parental lines mainly under salt stress, but also in the control conditions. Moreover, other post-germination traits (such as taller plants and greener leaves) presented an improvement in the related hybrids (Zhang *et al.*, 2021). In a rice hybrid, heterosis for salinity tolerance was characterized, by testing the hybrid and the parents at the 2-week-old seedling stage. The study concluded that the hybrid shows a higher survival rate after salinity treatment: 40.3% and 17% higher than the paternal and maternal lines, respectively (Liu *et al.*, 2024b).

Overall, future breeding strategies should consider not only the genetic factors influencing seed development (such as size, germination, and performance), but also their interaction with environmental factors and other variables.

## Conclusion and further perspectives

Recently, chemically induced epimutagenesis has emerged as a powerful tool to bypass hybrid vitality issues in seeds. As demonstrated by Huc *et al.* (2022), 5-Azacytidine, a DNA methyltransferase inhibitor, was used to treat Arabidopsis plants during early growth stages. This effectively led to hypomethylation in the CG context of the genome. This interesting biotechnological tool was used to reduce the expression of paternally expressed genes that are associated with hybrid seed failure (e.g. triploid block), allowing seeds to develop normally. The authors discovered that hypomethylation effects can be inherited transgenerationally, allowing the bypassing of hybrid vitality to persist beyond the treated generation (Huc *et al.*, 2022). This strategy could facilitate long-term breeding strategies without repeated chemical treatments, and could be a useful tool for broader application in other crop species. In fact, 5-Azacytidine can be applied in crosses between other crop species to enhance breeding programs; for example, to generate viable hybrids with desirable traits from crosses with wild relatives, or individuals or species with different ploidy levels (Huc *et al.*, 2022).

This is not a unique case, as in recent years, other chemicals have been used with similar purposes. For instance, zebularine was used as a DNA methylation inhibitor with multiple applications in plant epigenetics (Baubec *et al.*, 2009; Xue *et al.*, 2012). Trichostatin A (TSA), a histone deacetylase inhibitor, was used as a facilitating tool in the doubled haploid system, where some wheat genotypes and F_1_ hybrids exhibit low culture responses, yielding few or no embryos from microspores. TSA treatments improved doubled haploid responses in several ranges of spring and winter wheat genotypes (Jiang *et al.*, 2017). In *Tanacetum parthenium* (feverfew), a medicinal herb valued for its therapeutic compound parthenolide, specific concentrations of TSA significantly enhanced the expression of *GERMACRENE A SYNTHASE* (*GAS*) and *PARTHENOLIDE SYNTHASE* (*PTS*) genes, resulting in increased parthenolide accumulation (Alimirzaee *et al.*, 2024). BIX-01294, a histone methylation inhibitor, was shown to effectively regulate H3K9 methylation, a repressive epigenetic mark, in *Brassica napus* and *Hordeum vulgare* (Berenguer *et al.*, 2017). By down-regulating H3K9 methylation, BIX-01294 promotes microspore reprogramming and embryogenesis initiation, while higher methylation levels support later embryogenesis stages and activate key regulatory enzymes. Recently, the potentiality of BIX-01294 pharmacological treatments to enhance microspore embryogenesis efficiency has also been tested in recalcitrant plant species like bread wheat (Valero-Rubira *et al.*, 2024). Good target crops for breeding using epimutagenesis could be: (i) rice (*Oryza* spp.), where hybridization between *Oryza sativa* (domesticated rice) and wild relatives (e.g. *Oryza rufipogon*) often faces hybrid seed lethality (therefore, an epimutagenesis could facilitate introgression of traits like stress resistance from ‘wild’ species into ‘high-yielding’ varieties); (ii) potato (*Solanum tuberosum*), where ploidy barriers exist among wild and domesticated species, and overcoming barriers in interploidy crosses between diploid wild potatoes and tetraploid cultivated potatoes could help to transfer traits like pest resistance; (iii) barley (*Hordeum vulgare*), where this may facilitate crosses between diploid *Hordeum* species and cultivated tetraploid barley, to introduce resilience to salinity or drought; (iv) wheat (*Triticum aestivum*), where hybridization between polyploid wheat species (like tetraploid *Triticum turgidum* and hexaploid *Triticum aestivum*) and wild relatives often encounters reproductive barriers due to ploidy differences or incompatibility at the seed endosperm level. In this case, the use of epimutagenesis to transiently suppress DNA methylation could then bypass wheat endosperm-based hybridization barriers, allowing introgression of valuable traits like disease resistance, drought tolerance, or enhanced nutritional quality from diploid wild relatives into commercial wheat varieties. Since the methylation state recovers in subsequent generations, natural endosperm balance can stabilize the hybrid wheat seed, ensuring robust crop performance.

Overall, these approaches provide enhanced flexibility in breeding by transiently overcoming specific hybridization barriers without inducing permanent alterations to the epigenome. As this reversible epigenetic silencing can be undone, once the hybrid is stabilized, normal epigenetic mechanisms can be reestablished, allowing for the restoration of crucial agronomic traits influenced by methylation, such as stress tolerance (i.e. drought and salinity adaptation) and other adaptive responses (i.e. pathogen resistance and nutrient uptake efficiency). Initiatives focusing on model species are essential for bridging basic and applied research. The 1001 Genomes Project, a large-scale endeavor centered on *Arabidopsis thaliana*, exemplifies this approach (https://1001genomes.org/). Launched in 2008, the project aimed to sequence the genomes of over 1000 Arabidopsis accessions to provide comprehensive insights into its genetic diversity. This extensive dataset is invaluable for plant geneticists and breeders, facilitating the identification of genes associated with various traits, including those linked to heterosis. Further efforts are needed to incorporate additional aspects discussed here, such as the influence of environmental interactions (e.g. those discussed on heat, drought, and salinity stress in hybrid vigor), and the role of the microbiome (both studying rhizosphere and seed endophytic microbiomes), including its possible inheritance effect across generations.

Expanding the genetic diversity in breeding programs through wild relatives and neglected crops can help uncover novel heterotic effects, including at the transgenerational level. Additionally, advanced technologies, such as hybridization, seed propagation using clonal propagation tools, and the use of epigenetic silencers to mitigate genetic incompatibility in certain generations, are offering new opportunities. Alongside the already established use of synthetic biology and functional studies, adapting modern genome editing techniques offers next-generation tools for molecular breeding in novel hybrids.

## Data Availability

No new data were created in this study
